# *Trypanosoma cruzi* Evades the Protective Role of Interferon-Gamma-Signaling in Parasite-Infected Cells

**DOI:** 10.1371/journal.pone.0110512

**Published:** 2014-10-23

**Authors:** Philipp Stahl, Volker Ruppert, Ralph T. Schwarz, Thomas Meyer

**Affiliations:** 1 Institut für Virologie, AG Parasitologie, Philipps-Universität Marburg, Marburg, Germany; 2 Klinik für Kardiologie, Philipps-Universität Marburg, Marburg, Germany; 3 Klinik für Psychosomatische Medizin und Psychotherapie, Georg-August-Universität Göttingen, Göttingen, Germany; 4 Unité de Glycobiologie Structurale et Fonctionnelle, UMR CNRS/USTL n° 8576, Université de Lille1 Sciences et Technologies, Villeneuve d'Ascq, France; Karolinska Institutet, Sweden

## Abstract

The protozoan parasite *Trypanosoma cruzi* is responsible for the zoonotic Chagas disease, a chronic and systemic infection in humans and warm-blooded animals typically leading to progressive dilated cardiomyopathy and gastrointestinal manifestations. In the present study, we report that the transcription factor STAT1 (*s*ignal *t*ransducer and *a*ctivator of *t*ranscription *1*) reduces the susceptibility of human cells to infection with *T. cruzi*. Our *in vitro* data demonstrate that interferon -γ (IFNγ) pre-treatment causes *T. cruzi*-infected cells to enter an anti-parasitic state through the activation of the transcription factor STAT1. Whereas stimulation of STAT1-expressing cells with IFNγ significantly impaired intracellular replication of parasites, no protective effect of IFNγ was observed in STAT1-deficient U3A cells. The gene encoding indoleamine 2, 3-dioxygenase (*ido*) was identified as a STAT1-regulated target gene engaged in parasite clearance. Exposure of cells to *T. cruzi* trypomastigotes in the absence of IFNγ resulted in both sustained tyrosine and serine phosphorylation of STAT1 and its increased DNA binding. Furthermore, we found that in response to *T. cruzi* the total amount of intracellular STAT1 increased in an infectious dose-dependent manner, both at the mRNA and protein level. While STAT1 activation is a potent strategy of the host in the fight against the invading pathogen, amastigotes replicating intracellularly antagonize this pathway by specifically promoting the dephosphorylation of STAT1 serine 727, thereby partially circumventing its protective effects. These findings point to the crucial role of the IFNγ/STAT1 signal pathway in the evolutionary combat between *T. cruzi* parasites and their host.

## Introduction

*Trypanosoma cruzi* is the causative agent of Chagas disease, an endemic infection in Latin America characterized in the chronic stage by dilated cardiomyopathy and megavisceral syndromes. The disease is transmitted to humans through hematophagous insect vectors called triatomines, which are members of the *Reduviidae* family and the *Triatominae* subfamily. The complex life cycle of *T. cruzi* begins when a triatomine ingests flagellated trypomastigotes during a blood meal from an infected mammalian host [1]–[3]. The parasite then passes through the triatomine digestive tract and undergoes a number of morphological differentiations that result in the production and multiplication of epimastigote parasites. During the next blood meal, the insect excretes highly infective metacyclic trypomastigotes via faeces, and, subsequently, the parasites can invade their new host directly through the vector's bite or by crossing mucous membranes. After host cell penetration, which coincides with the formation of a nascent parasitophorous vacuole, the parasite is released in the cytoplasm where numerous amastigotes are formed through binary fission [4]. The intracellular amastigotes then transform into trypomastigotes, which burst out of the cell, enter the bloodstream, and disseminate throughout the host. The newly infected host can then serve as a reservoir for further parasite propagation. However, non-vectorial mechanisms of infection have also been identified, such as congenital transmission, blood transfusion, organ transplantation, and recently incidental ingestion of parasite-contaminated food or drink [5].

Both innate and acquired immune responses are crucial for controlling *T. cruzi* dissemination and host survival [6]. The transmembrane *T*oll-*l*ike *r*eceptor (TLR) family of *p*attern *r*ecognition *r*eceptors (PRRs) plays an important role in the recognition of *T. cruzi* during early infection. Binding of DNA, RNA or *g*lycosyl*p*hosphatidyl-*i*nositol (GPI) anchors from trypomastigotes to distinct members of the TLR family initiates a signaling cascade that is dependent on the adaptor molecule *my*eloid *d*ifferentiation factor 88 (MyD88) and culminates in the activation of pro-inflammatory genes crucial for the resistance to *T. cruzi* infection, such as interleukin-1β (IL-1β) [7], IL-6 [7], [8], IL-12 [9]–[13], *t*umor *n*ecrosis *f*actor-*α* (TNFα) [10], [11], [14], interferon-β (IFNβ) [15]–[18], and interferon-γ (IFNγ) [7], [12], [13], [16], [19], [20]. Mice lacking functional MyD88 are highly susceptible to *T. cruzi* infection, possibly because of defects in the production of pro-inflammatory cytokines [21]. In *T. cruzi*-infected macrophages, gene expression of pro-inflammatory cytokines is mediated by the two transcription factors *n*uclear *f*actor-*κB* (NF-κB) [22]–[24] and *i*nterferon-*r*egulatory *f*actor 3 (IRF3) [18]. NF-κB additionally activates the *i*nducible *n*itric *o*xide *s*ynthase (iNOS), which catalyzes the production of microbicidal *n*itric *o*xide (NO). Mice deficient in iNOS or the IFNγ receptor are highly susceptible to *T. cruzi* infections with increased parasite burdens and their macrophages show impaired trypanocidal activities due to a lack of NO production [25].

While the important role of MyD88- and TRIF-dependent signal pathways for the pathogenesis of Chagas disease is well established, much less is known about the contribution of STAT proteins (*s*ignal *t*ransducer and *a*ctivator of *t*ranscription) to the control of *T. cruzi* infection [24], [26]–[28]. The founding member of this family of cytokine-driven transcription factors, STAT1, has been reported to be an important anti-microbial mediator of host resistance to *Toxoplasma gondii*, but data on *T. cruzi* infection are controversial [27], [29], [30]. Upon binding of IFNγ to its receptor, non-covalently receptor-associated *Ja*nus *k*inases (JAKs) phosphorylate the cytoplasmic tail of the IFNγ receptor, thereby permitting recruitment of non-phosphorylated STAT1 (for a review see, e.g. [31]–[33]). In the next step, the activated JAKs then phosphorylate STAT1 on a signature tyrosine residue (Y701) in its carboxy-terminus. Tyrosine-phosphorylated STAT1 dimers are then translocated to the nucleus, where they bind to palindromic *g*amma-*a*ctivated *s*ites (GAS) in the promoter regions of IFNγ-responsive genes to initiate transcription. Phosphorylation of serine 727 induces nuclear export acceleration [34] which is required for full-fledged transcriptional activation [35]–[37].

In our study, we sought to investigate the impact of the IFNγ/STAT1 pathway on *T. cruzi* infection in human cells. Our *in vitro* data demonstrate that *T. cruzi* multiplication resulted in both infectious dose-dependent STAT1 serine 727 and tyrosine 701 phosphorylation which was associated with increased binding to GAS elements. In addition, we revealed that STAT1 signaling functions as a protective factor but is also subjected to inhibition by the parasite.

## Materials and Methods

### Cell lines, transfection, and parasite culture

Human foreskin fibroblasts (HFF, obtained from American Type Culture Collection [ATCC], Manassas, USA), adenocarcinomic alveolar basal epithelial cells (A549, ATCC), Vero E6 cells (ATCC) and STAT1-negative fibrosarcoma cells (U3A) [38] were grown in Dulbecco's modified eagle medium (DMEM; Gibco) supplemented with 10% fetal calf serum (FCS; Biochrom), 1% penicillin, and 1% streptomycin. Cells were cultivated at 37°C in a humidified 5% CO_2_ atmosphere in cell culture flasks and passaged by trypsinization. Parasites from the CL Brener and Brazilian Y strain, a gift from Dr. M. A. Campos (Research Centre René Rachou, Fiocruz, Belo Horizonte, Brazil) and Dr. T. Jacobs (Bernhard-Nocht-Institut für Tropenmedizin, Hamburg, Germany), were grown in Vero E6 cells in DMEM supplemented with 1% fetal calf serum. Motile and infective trypomastigotes were collected from the culture supernatant, washed twice with phosphate-buffered saline (PBS) and were either used for experiments or propagation of the parasite culture. For stimulation of human cells, IFNγ (Biomol) was used at a concentration of 5 ng/ml. The indoleamine 2,3-dioxygenase (IDO) inhibitor 1-methyltryptophan (1-MT, Sigma-Aldrich) was used at a final concentration of 1.5 mM, and the potent iNOS inhibitor S-methylisothiourea sulfate (SMT, Fluka Analytics) was used at 1 mM. The inhibitors were added to HFF cells together with IFNγ and, after 24 h of co-incubation, cells were washed twice with PBS. *T. cruzi* trypomastigotes were then added together with fresh culture medium at a cell-to-parasite ratio of 1∶2 and removed after 24 h of incubation. Culture supernatants were removed 72 h p.i., and the fixed cells were stained for light microscopy. In a subset of experiments, the JAK inhibitor AG-490 was added at a concentration of 50 µM. For dephosphorylation assays, U3A cells were transfected with the pSTAT1-GFP vector coding for a carboxy-terminal fusion protein of full-length human STAT1 with *g*reen *f*luorescent *p*rotein (GFP), while for gel shift experiments U3A cells were reconstituted with recombinant untagged STAT1 using the plasmid pSTAT1 [39]. For fluorescence microscopical detection of *T. cruzi*, U3A cells were reconstituted with plasmids coding for wild-type STAT1 (pSTAT1-WT-GFP) or its tyrosine-phosphorylation-deficient point mutant Y701F (pSTAT1-Y701F-GFP). Transfection was performed using MegaTran1.0 (Origene) according to the manufacturer's recommendation. Twenty-four hours after transfection, cells were stimulated with 5 ng/ml of recombinant human IFNγ.

### Cell infection and analysis of infectivity

Confluent HFF or U3A cells were either left untreated or treated with IFNγ (5 ng/ml) for 6, 12 or 24 h, as indicated, before the cells were infected with trypomastigotes with a multiplicity of infection (MOI) of 2 or 10. After 24 h of infection, remaining parasites in the culture medium that had not infected cells were removed by washing, and infected cells were replaced in fresh DMEM for an additional 48 h. The cells were then fixed with ice-cold methanol and stained with crystal violet and May-Grünwald (both from Merck). The mean numbers of infected cells and intracellular amastigotes per cell were blindly counted in 10 non-overlapping high power microscopic fields in three independent experiments performed in duplicate.

### Determination of IDO enzymatic activity

To measure enzymatic activity of indoleamine 2,3-dioxygenase (IDO), HFF cells grown in 96-well microtiter plates in DMEM containing 5% FCS supplemented with 0.6 mM L-tryptophan were either treated for 72 h with IFNγ or left untreated. Then 160 µl of media were removed from each well and transferred to a 96-well V-bottomed plate. Enzymatic activity of IDO was determined spectrophotometrically by measuring the concentration of kynurenine, which directly correlates to the IDO-produced concentration of N-formyl-kynurenine [40]. After addition of 10 µL 30% trichloroacetic acid to each well, the plates were incubated at 50°C for 30 min to hydrolyze N-formyl-kynurenine to kynurenine and then centrifuged at 500 g for 10 min. One hundred µL of each culture supernatant were transferred to 96-well flat-bottomed plates and mixed with an equal volume of Ehrlich reagent (1.2% (w/v) p-dimethylaminobenzaldehyde in glacial acetic acid). After leaving for 10 min at room temperature, the extinction was determined at 492 nm using a microplate reader (BioTek). Experiments were performed in triplicates, and data are presented relative to those of unstimulated samples, set as 100.

### Measurement of nitric oxide

Nitric oxide (NO) production by untreated and IFNγ-treated confluent HFF cells was determined by measuring the nitrite concentration in the culture media. Briefly, 100 µl of medium were mixed with an equal volume of Griess reagent (1 part 0.1% naphthylethylenediamine dihydrochloride to 1 part 1% sulfanilamide in 5% phosphoric acid) and absorbance was measured at 540 nm after 10 minutes incubation in the dark.

### Immunocytochemistry

Adherent HFF and U3A cells grown on 8-well chamber slides were either left uninfected or incubated with *T. cruzi* parasites for 18 h. Cells were fixed with methanol at −20°C for 20 min and, after two washes in PBS, permeabilized with 1.0% Triton X-100 in PBS for 20 min. Non-specific binding was blocked by incubation with 25% FCS/PBS for 45 min at RT before the samples were incubated for 45 min with anti-STAT1 antibody C-24 (Santa Cruz) diluted 1∶1000 in 25% FCS/PBS. After three washes in PBS, the specimens were incubated with Cy3-conjugated secondary antibody (Dianova), diluted 1∶500 in PBS, for an additional 45 min. Nuclei of human cells and intracellular parasites were detected by staining with Hoechst 33258 dye at a final concentration of 5 µg/ml. Samples were mounted in fluorescence mounting medium (Southern Biotech) and visualized using an Axiovert 200 M microscope (Carl Zeiss) equipped with appropriate fluorescence filters. Images were obtained with a CCD camera and further processed with the Image-Pro MDA5.1 (Media Cybernetics) software. For microscopic localization of GFP-tagged STAT1, cells were fixed with 4% paraformaldehyde in PBS and stained with Hoechst dye.

### Cell lysis

Mock-infected and *T. cruzi*-infected cells grown on 6-well dishes were lysed for 5 min on ice in 50 µl cytoplasmic extraction buffer (20 mM Hepes, pH 7.4, 10 mM KCl, 10% (v/v) glycerol, 1 mM EDTA, 0.1 mM Na_3_VO_4_, 0.1% IGEPAL-CA-360, 3 mM DTT, 0.4 mM Pefabloc, Complete Mini protease inhibitors (Roche)). The lysates were centrifuged at 16000 g (15 sec, 4°C), and supernatants spun again for 5 min at 16000 g. The supernatants resulting from this centrifugation step were collected as cytoplasmic extracts, while the pellets were resuspended in 50 µl nuclear extraction buffer (20 mM Hepes, pH 7.4, 420 mM KCl, 20% (v/v) glycerol, 1 mM EDTA, 0.1 mM Na_3_VO_4_, 3 mM DTT, 0.4 mM Pefabloc, and Complete Mini protease inhibitors) and left on ice for 30 min. The nuclear extracts were spun at 16000 g and 4°C for 15 min. The supernatants of this centrifugation step were mixed with cytoplasmic extracts to obtain whole cell extracts, which were used for Western blotting, electrophoretic mobility shift assays (EMSAs) and *in vitro* dephosphorylation assays.

### Western blotting

For immunoblotting experiments, confluent cells grown in 6-well tissue culture plates in DMEM containing 10% FCS were incubated with IFNγ (5 ng/ml) and/or infected with trypomastigotes at different MOIs or left untreated. Supernatants were removed after 18 h and cells washed thrice with PBS. After cell fractionation, the combined cytoplasmic and nuclear lysates were boiled in SDS sample buffer and resolved by 10% SDS-PAGE with subsequent transfer onto PVDF membranes. The membranes were incubated first with a phospho-Tyr701-specific STAT1 antibody (Cell Signaling) and then with a conjugated anti-rabbit secondary antibody (Li-Cor). To determine the amount of total STAT1, blots were stripped for 60 min at 60°C in a buffer containing 2% SDS, 0.7% β-mercaptoethanol, and 62.5 mM Tris-HCl, pH 6.8 and then successively re-probed with phospho-Ser727-antibody and the pan-STAT1 polyclonal antibody C-24 (both rabbit antibodies from Santa Cruz Biotechnology), the latter reacting with both phosphorylated and non-phosphorylated STAT1. Bound immunoreactivity was detected with secondary IRDye 800CW antibodies visualized on a Li-Cor Odyssey imaging machine.

### *In vitro* dephosphorylation assay

To assess whether *T. cruzi*-infected cells express phosphatase or protease activity specifically targeting endogenous STAT1, *in vitro* dephosphorylation assays using recombinant STAT1-GFP as substrate were performed. Briefly, equal amounts of cell extracts from uninfected STAT1-GFP-expressing U3A cells (20 µl) were mixed with extracts from *T. cruzi*-infected or mock-treated STAT1-negative U3A cells and incubated for either 0 min or 45 min at room temperature. Samples were boiled in SDS sample buffer and monitored for serine and tyrosine phosphorylation by means of Western blotting.

### Electrophoretic mobility shift assay

To probe for STAT1 DNA-binding activity in *T. cruzi*-infected cells, electrophoretic mobility shift assays (EMSAs) were performed. Five microliters of cellular extracts were incubated with 1 ng of [^33^P]-labeled M67 duplex oligonucleotide [39]. The radioactively labeled probe, which was generated by an end-filling reaction using the Klenow fragment (New England Biolabs), contained a consensus STAT1-binding site (5′-CGACATTTCCCGTAAATCTG-′3; GAS sequence underlined, 4 bp overhangs at the 5' end and the respective antisense oligo are not shown). For competition experiments, cellular extracts were first incubated with [^33^P]-labeled M67 in EMSA buffer for 15 min at RT, and then a 750-fold molar excess of unlabeled M67 DNA was added. In supershift assays, 20 ng of either the STAT1-specific antibody C-24 or a non-specific STAT3 antibody were present in the shift reaction. The reactions were loaded on a 4% 29∶1 acrylamide∶bisacrylamide gel at 4°C and separated at 400 V. DNA-binding activity of STAT1 was visualized on vacuum-dried gels with a phosphoimaging system (FLA-5100, Fuji) using the programs Aida Image Analyzer v.4.06 and TINA 2.0 (Raytest).

### Analysis of mRNA expression

Cells were incubated for 18 h with medium alone, with *T. cruzi* trypomastigotes, or IFNγ. Total RNA was extracted from the cells by using the PeqGold Total RNA kit (Peqlab), according to the manufacturer's instructions. The High Capacity cDNA Reverse Transcription Kit (Applied Biosystems) was used to convert mRNA into cDNA at 42°C for 120 min and followed by a denaturation step at 95°C for 2 min. Amplification was then performed by *p*olymerase *c*hain *r*eaction (PCR) in an I-cycler (Bio-Rad) using SsoAdvanced SYBR Green Supermix (Bio-Rad) and the primer pairs indicated in File S1. The following PCR program was applied: initial denaturation at 95°C for 30 sec followed by 40 cycles of denaturation at 95°C for 5 sec and annealing/extension at 55°C for 20 sec. A melting curve analysis was run after final amplification via a temperature gradient from 55 to 94°C in 0.5°C increment steps measuring fluorescence at each temperature for a period of 10 s. All reactions were carried out in duplicate for each sample. Using the Bio-Rad software, the threshold (C_t_) at which the cycle numbers were measured was adjusted to areas of exponential amplification of the traces. The ΔΔC_t_ method was used to compare expression levels of two samples by applying the formula 2^−(ΔC^_t_^ target − ΔC^_t_^ reference sample)^, with *gapdh* as control [41].

### Statistical analyses

Means and standard deviations were calculated for each infection and stimulation mode. Differences in infection status as well as STAT1 phosphorylation level and DNA-binding activity were assessed using Student's *t* tests and Mann-Whitney-Wilcoxon tests, where appropriate. Data were analyzed using the Sigmastat (Systat Software) program. In all analyses, a p value ≤0.05 was used to indicate statistical significance.

## Results

### STAT1 is required for IFNγ-induced protection against *T. cruzi* infection

**Figure 1:**
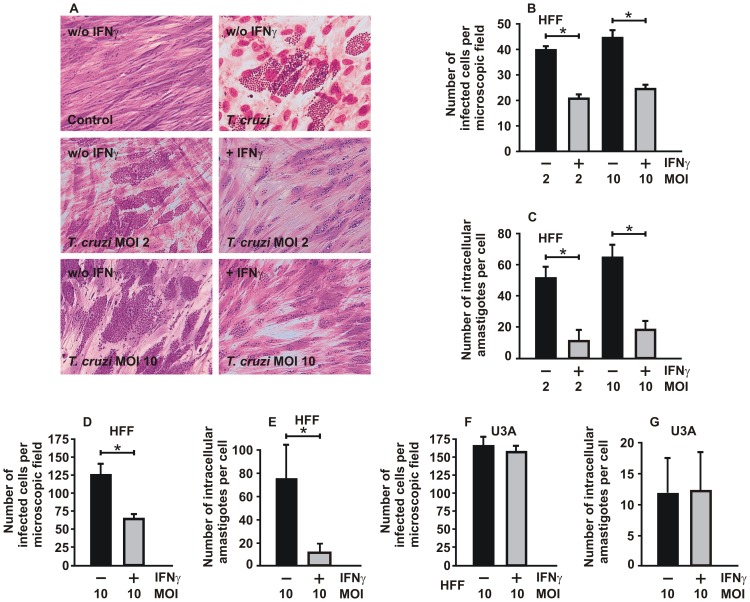
STAT1 deficiency in U3A cells is associated with high susceptibility to *T. cruzi* infection and impaired cellular responses to IFNγ. (A) Stimulation of HFF cells with IFNγ (5 ng/ml for 6 h) resulted in reduced numbers of intracellular *T. cruzi* amastigotes as determined by conventional crystal violet and May-Grünwald staining. As indicated, cells were either left untreated (w/o IFNγ) or treated with IFNγ (+IFNγ) before being infected with trypomastigotes of the Brazilian Y strain of *T. cruzi* at MOIs of 0, 2 and 10, respectively (n = 2 in duplicate). (B-E) The histograms depict the average number of infected cells per microscopic field (B,D) and the average number of intracellular amastigotes per cell (C,E) in untreated HFF cells (black columns) and cells pre-treated with IFNγ (grey columns) for 6 h (B,C) and 24 h (D,E), respectively. Fibroblasts were infected with parasites at 2 or 10 MOI before parasite burden was quantified microscopically. (F,G) No inhibitory effects of IFNγ pre-treatment on *T. cruzi*-infected U3A cells lacking STAT1 expression. Similar experiment with respect to stimulation of cells with IFNγ, incubation with parasites and measurement of parasite invasion as described in (D,E), except that U3A cells were used.

To assess the role of STAT1 transcription factor in IFNγ-dependent protection against infection by *T. cruzi*, we first performed invasion assays using trypomastigotes in HFF cells expressing endogenous STAT1 and in STAT1-negative U3A cells, both in the absence and presence of IFNγ. The data showed that infection of HFF cells by both the pathogenic Y strain and the low virulent CL Brener strain of *T. cruzi* resulted in high invasion and multiplication indices over 72 h, as determined by microscopic examination using crystal violet and May-Grünwald stainings (Fig. 1A). We confirmed that both the percentages of infected cells and the numbers of intracellular amastigotes per cell were significantly higher in untreated HFF cells as compared to cells which had been pre-treated for 6 h with 5 ng/ml of IFNγ before exposure to parasites (Fig. 1B,C). Similar results were observed when the incubation time was extended to 12 h (Fig. S1A,B in File S1) or 24 h (Fig. 1D,E), except that the protective effect of IFNγ was more pronounced. The inhibitory effect of IFNγ on parasite replication required pre-treatment of cells with the cytokine (for 6 h) and was not observed when cells were exposed simultaneously to IFNγ and parasites (co-incubation, Fig. S1C,D in File S1). As demonstrated in Figure 1D and F, the mean number of *T. cruzi*-infected cells per microscopic field was higher in U3A cells than in HFF cells. Since U3A cells derived from a human fibrosarcoma typically displayed a smaller cytoplasmic volume than non-tumorigenic HFF cells, the average number of intracellular amastigotes was higher in the latter cell line. However, when U3A cells lacking endogenous STAT1 expression were substituted for HFF cells, we found that the added cytokine no longer elicited beneficial effects with respect to the resistance to *T. cruzi* infection. In particular, neither the number of infected cells (Fig. 1F) nor the number of replicating amastigotes (Fig. 1G) was significantly reduced upon pre-treatment with a high dose of IFNγ. Thus, treatment with IFNγ had no inhibitory effect on parasite growth most probably due to the STAT1 deficiency of this cell line.

**Figure 2:**
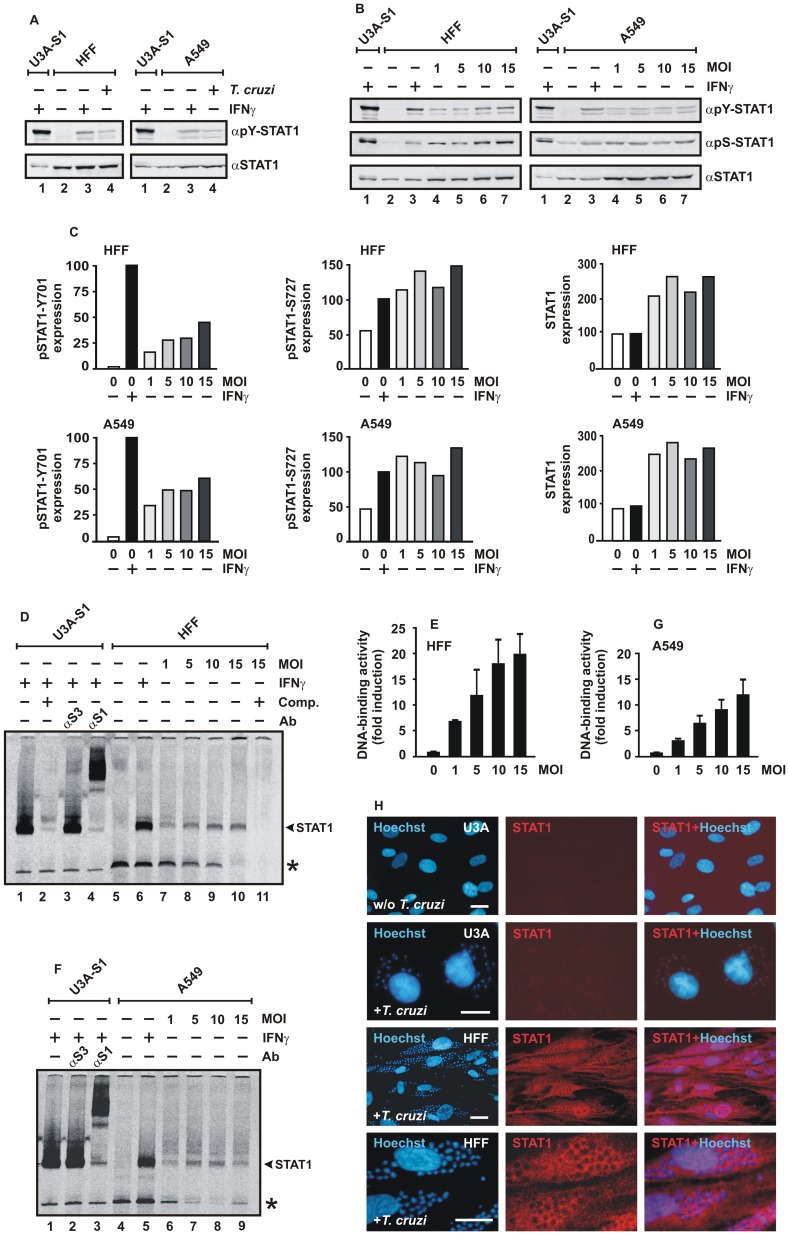
Parasitic infection with *T. cruzi* induces a sustained and cell type-independent activation of STAT1. (A) Immunoblots demonstrating tyrosine phosphorylation and expression levels of STAT1 in differentially treated HFF (left) and A549 cells (right) using antibodies against tyrosine-phosphorylated STAT1 (αpY-STAT1) and, after stripping off of bound immunoreactivity from the membranes, pan-STAT1 antibody (αSTAT1). Cells were either left untreated (lane 2), stimulated for 18 h with 5 ng/ml IFNγ (lane 3) or challenged for 18 h with *T. cruzi* trypomastigotes at an MOI of 10 (lane 4). Cellular extracts from IFNγ-treated U3A cells (45 min) expressing recombinant STAT1 were used as control (lane 1) (n = 3). (B) Infectious dose-dependent increase in STAT1 tyrosine and serine phosphorylation in cytokine-unstimulated HFF (left) and A549 cells (right). Equal numbers of cells were exposed to parasites for 18 h at different MOIs. Representative immunoblotting experiments using antibodies specifically reacting with phospho-Y701- (αpY-STAT1), phospho-S727- (αpS-STAT1) and pan-STAT1 antibody (αSTAT1) are shown (n = 4). (C) Quantification of Western blot results for expression of phospho-Y701-, phospho-S727- and total STAT1 in extracts from cells infected with increasing doses of *T. cruzi*, as depicted in Figure 2B. Phosphorylation and expression levels were compared to the signal intensity in uninfected cells stimulated for 3 h with IFNγ set as 100. (D-G) *T. cruzi* infection elicits GAS-binding activity. Similar extracts as used for Western blotting (B) were incubated for 30 min with [^33^P]-labeled DNA containing a single STAT binding site (M67) and then loaded onto a polyacrylamide gel for detection by EMSA. (D,F) The band corresponding to M67-bound STAT1 dimers was identified by supershift with a STAT1-, but not STAT3 antibody (Ab) and, in addition, by competition with a 750-fold molar excess of unlabeled M67-DNA (Comp.). STAT1-M67 complexes are marked with an arrowhead, asterisks indicate an unspecific band. (E,G) The histograms demonstrate the M67-binding activity in cytokine-untreated HFF cells (E) and A549 cells (G) plotted against the infection dose (MOI). (H) Immunocytochemical staining of STAT1 in *T. cruzi*-infected HFF cells using anti-STAT1 antibody C-24 and Cy3-labeled secondary antibody. The fluorescence micrographs show the intracellular distribution of endogenous STAT1 in methanol-fixed, Hoechst-stained HFF cells and, in contrast, the lack of STAT1 expression in uninfected (w/o *T. cruzi*) and infected (+*T. cruzi*) U3A cells (scale bar 20 µm). Note that there was no co-localization of cytoplasmic STAT1 and Hoechst-stained amastigotes and that some parasite-containing cells showed nuclear accumulation of STAT1.

To exclude the possibility that this negative finding resulted from defective IFNγ receptor activation, we reconstituted U3A cells with STAT1 and subsequently stimulated the transfected cells with 5 ng/ml of IFNγ. As expected, cytokine stimulation of STAT1-reconstituted U3A cells induced tyrosine phosphorylation of the recombinant STAT1 (Fig. 2A,B) and resulted in sequence-specific DNA binding (Fig. 2D,F), confirming that U3A cells consisted of all components of an intact IFNγ signal pathway with the exception of STAT1. Together, the infection experiments in two cultured human cell lines demonstrated that IFNγ is engaged in the protection from *T. cruzi* infection and, moreover, point to a role of STAT1 in this process.

### *T. cruzi* infection leads to phosphorylation and GAS binding of STAT1

Given the fact that IFNγ has lost its anti-microbial effects in *T. cruzi*-infected STAT1-negative U3A cells, we examined, in more detail, the vital impact of STAT1 in counteracting parasite invasion. As shown in Figure 2A, IFNγ stimulation of STAT1-reconstituted U3A cells induced tyrosine phosphorylation of the recombinant STAT1. In addition, immunoblotting experiments showed that 18-hours exposure of STAT1-expressing cells to high doses of IFNγ resulted in a sustained phosphorylation of the critical tyrosine residue 701, which is required for the formation of transcriptionally active homodimers (Fig. 2A). Tyrosine phosphorylation was observed in IFNγ-stimulated mesenchymal HFF cells as well as epithelial A549 cells. Moreover, tyrosine phosphorylation was also detected in *T. cruzi*-infected cells in the absence of any IFNγ added to the culture media. While cellular lysates from untreated HFF and A549 cells showed no detectable STAT1 tyrosine phosphorylation, there was notable phosphate incorporation at residue 701 in *T. cruzi*-infected cells, which was clearly above the detection threshold as demonstrated by Western blotting. However, in infected HFF and A549 cells, the amount of tyrosine-phosphorylated STAT1 was substantially lower as compared to STAT1-reconstituted U3A cells stimulated with IFNγ for 45 min to achieve maximal levels of tyrosine-phosphorylated STAT1. After 6 h of continuous exposure to IFNγ, the phospho-tyrosine signal had ceased in non-infected cells, whereas *T. cruzi*-infected HFF cells still exhibited a strong signal (Fig. S2 in File S1).

Next, we tested whether adding increasing parasite numbers leads to elevated levels of STAT1 tyrosine phosphorylation. For this purpose, we used cellular extracts from an equal number of cells infected with increasing parasite titers and probed for the amount of tyrosine- and serine-phosphorylated STAT1 using antibodies specifically recognizing phosphoY701 and phosphoS727, respectively. As expected, the results demonstrated that increasing MOIs were associated with elevated levels of tyrosine-phosphorylated STAT1. Furthermore, also the amount of serine-phosphorylated STAT1 increased in relation to the number of added parasites (Fig. 2B,C). Given that in IFNγ-stimulated cells phospho-STAT1 induces the up-regulation of its own gene at a transcriptional level [42], we were not surprised that due to this positive feed-back loop also the amount of total cellular STAT1 was significantly increased in an infectious dose-dependent manner.

Although weaker than in the case with IFNγ treatment of STAT1-reconstituted U3A cells, electrophoretic mobility shift assays still demonstrated a detectable DNA-binding activity to a GAS element in lysates from *T. cruzi*-infected HFF cells (Fig. 2D,E) as well as A549 cells (Fig. 2F,G). Similar to the results from the Western blot experiments, infection with *T. cruzi* alone induced STAT1 activation as demonstrated by binding to high-affinity GAS elements and, in addition, this binding activity correlated positively with parasite burden.

To assess the intracellular distribution of STAT1 in infected cells, immunocytochemical stainings using a pan-STAT1 antibody were performed. While U3A cells used as a negative control showed no detectable immunofluorescence signals, HFF cells infected with *T. cruzi* displayed positive immunoreactivity both in the cytosol and the nucleus (Fig. 2H). In numerous cells containing amastigotes, there was evidence of a nuclear accumulation of STAT1, as defined by significantly higher immunofluorescence intensities in the nuclear as compared to the corresponding cytoplasmic compartment. In general, cytoplasmic STAT1 localization was confined to areas free of replicating parasites.

### Intracellular *T. cruzi* parasites activate IFNγ-driven STAT1 target genes

**Figure 3:**
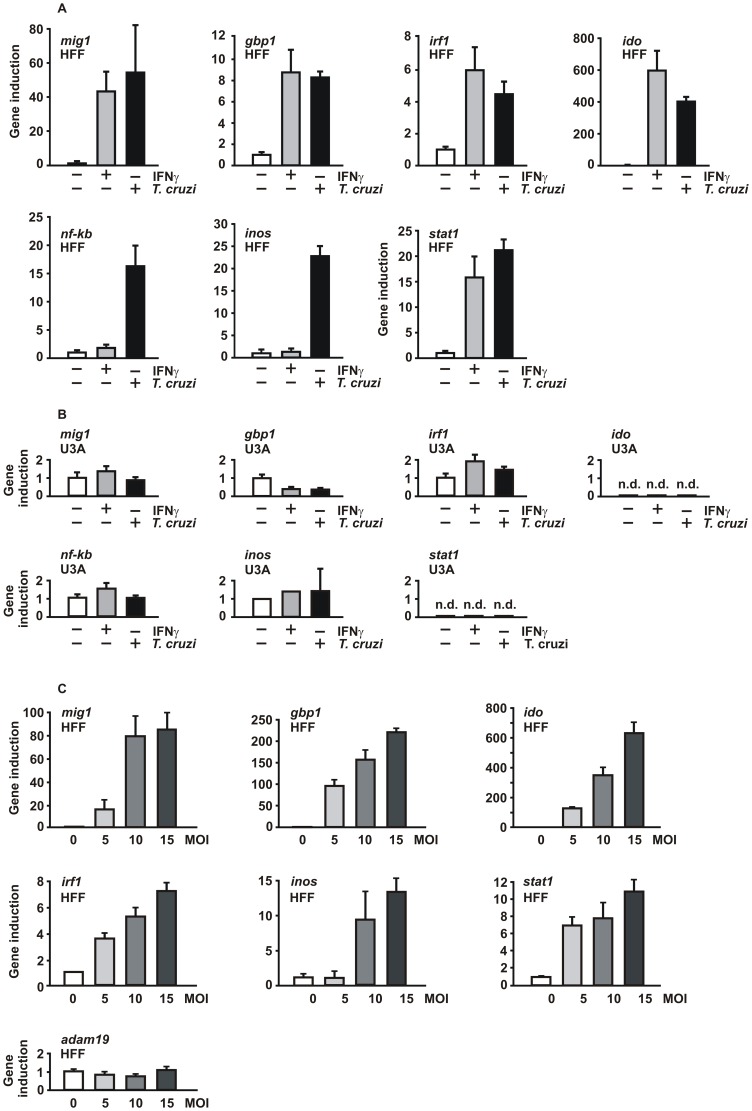
Induction of STAT1-regulated genes in *T. cruzi*-infected cells. (A) HFF cells were either left untreated, stimulated for 6 h with 5 ng/ml of IFNγ or infected for 18 h with *T. cruzi* parasites at an MOI of 10 in the absence of cytokine exposure, as determined by real-time PCR assays. Histograms depict expression levels of the *mig1*, *gbp1*, *irf1*, *ido*, *stat1*, *inos*, and *nf-kb* gene before (white columns) and after 6 h stimulation of cells with IFNγ (grey columns) as well as after parasite infection (black columns). Specific gene induction was normalized to the expression level of the housekeeping gene *gapdh*. The data are presented as means and standard deviations from at least three independent experiments. (B) U3A cells lacking STAT1 expression showed no induction of these genes under the same experimental conditions as used in (A) (n.d. =  not detectable). (C) Infectious dose-dependent increase in STAT1-regulated gene expression in *T. cruzi*-infected HFF cells. The *adam19* gene was used as a negative control (n = 3).

The experiments presented thus far have shown that incubation of two human cell lines of either fibroblast or epithelial origin with *T. cruzi* trypomastigotes was linked to an activation of STAT1, as shown by increased tyrosine and serine phosphorylation and binding to GAS elements. Taking into consideration that IFNγ stimulation of cells promotes STAT1-mediated gene expression, we sought to determine whether STAT1 functions as a transcription factor in the signaling pathway that is initiated by *T. cruzi* infection. To this end, we stimulated equal numbers of HFF cells for 6 h with 5 ng/ml IFNγ or infected them for 18 h with *T. cruzi* at an MOI of 10 in the absence of cytokine addition, before in cell lysates specific mRNA levels were measured by means of real-time PCR. As controls, we determined the transcript levels in HFF cells which had neither been treated with IFNγ nor infected with parasite and, in addition, used STAT1-negative U3A cells treated according to the protocol described above. In HFF cells, both IFNγ treatment and *T. cruzi* infection resulted in a robust activation of the STAT1-dependent target genes *mig1*, *gbp1*, *irf1*, *ido*, and *stat1*, whereas parasite infection alone, but not IFNγ, induced *inos* and *nf-kb* gene expression (Fig. 3A). However, no induction of these genes was observed in U3A cells, irrespective of whether they had been pre-treated with IFNγ or challenged with parasites (Fig. 3B).

To evaluate whether the induction of mRNA expression correlated with the parasite burden, we infected equal numbers of HFF cells with increasing MOIs ranging from 0 to 15 and subsequently measured transcript levels by RT-PCR. With the exception of *adam19* used as a negative control, all tested genes up-regulated by parasite infection showed a titer-dependent increase (Fig. 3C). Similar results were obtained in A549 cells (Fig. S3 in File S1). These data confirmed that STAT1 participates in the regulatory effects of IFNγ on *T. cruzi* parasitism.

**Figure 4:**
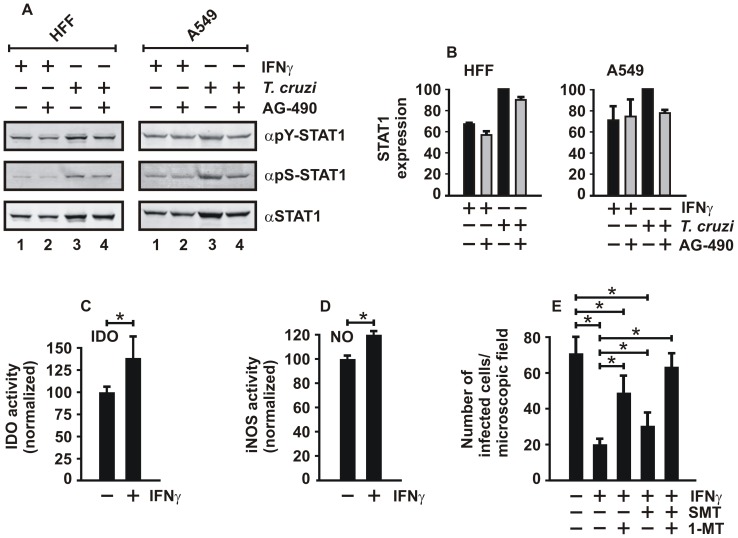
STAT1-regulated expression of *ido* is involved in the control of *T. cruzi* infection. (A,B) Exposure of cells to the JAK inhibitor AG-490 resulted in decreased STAT1 phosphorylation both at tyrosine residue 701 and serine residue 727 and is associated with a reduced intracellular STAT1 expression. Equal numbers of HFF and A549 cells were either pre-treated with 5 ng/ml of IFNγ or challenged for 18 h with parasites at an MOI of 15, in the absence or presence of AG-490 (50 µM). Representative Western blot results (A) and the corresponding quantification of STAT1 expression (B) are shown (n = 4). (C,D) Stimulation of HFF cells with IFNγ leads to increased enzymatic activity of indoleamine 2,3-dioxygenase (IDO, C) and elevated NO production (D), as measured with Ehrlich and Griess reagent, respectively (n = 3 in triplicate). (E) Inhibition of IDO by 1-methyltryptophan (1-MT, 1.5 mM) or iNOS by S-methylisothiourea sulfate (SMT, 1 mM) in IFNγ-pre-treated HFF cells resulted in significantly elevated numbers of *T. cruzi*-replicating cells (n = 4 in triplicate).

Next, the action of the JAK inhibitor AG-490, also termed tyrphostin B42, on *T. cruzi*-mediated STAT1 activation was studied by measuring its effect on tyrosine and serine phosphorylation. As shown in Figure 4, incubation of either HFF cells or A549 cells for 18 h with AG-490 at a concentration of 50 µM resulted in a significant reduction of both tyrosine- and serine-phosphorylated STAT1 as well as the total cellular amount of STAT1 (Fig. 4A,B). The impaired phosphorylation status was particularly observed in cells infected with parasites at an MOI of 15. This finding corroborated that pharmacological suppression of IFNγ-mediated signaling in cells challenged with parasites critically affected the activation of STAT1.

Based on our observation that infection with *T. cruzi* increased expression of the *ido* and *inos* gene titer-dependently (Fig. 3C), we next assessed the enzymatic activity of the two gene products in cytokine-stimulated HFF cells. The results showed that in cells stimulated with IFNγ for 72 h the synthesis of N-formyl-kynurenine from tryptophan catalyzed by IDO was increased as compared to untreated cells (Fig. 4C). Likewise, NO generation was enhanced as a consequence of IFNγ treatment (Fig. 4D).

In the light of the previous results, we also tested whether inhibition of either IDO or iNOS had any effect on parasite susceptibility. To this end, HFF cells were infected with *T. cruzi* trypomastigotes at an MOI of 1 for 72 h and the cells treated with the IDO blocker 1-methyltryptophan (1-MT, 1.5 mM) and the iNOS inhibitor S-methylisothiourea sulfate (SMT, 1 mM), respectively. Again, the invasion assay demonstrated that IFNγ pre-treatment of cells dramatically reduced the numbers of infected cells (Fig. 4E). However, exposure of IFNγ-treated cells to either 1-MT or SMT significantly blunted this effect, although no additive or synergistic effect was observed when the two inhibitors were added in combination to the cells. These results together suggested that the protective role of IFNγ treatment is in part executed by the action of the STAT1-regulated enzyme IDO, which is involved in parasite clearance.

### Evidence that *T. cruzi* infection directly impedes STAT1 signaling

**Figure 5:**
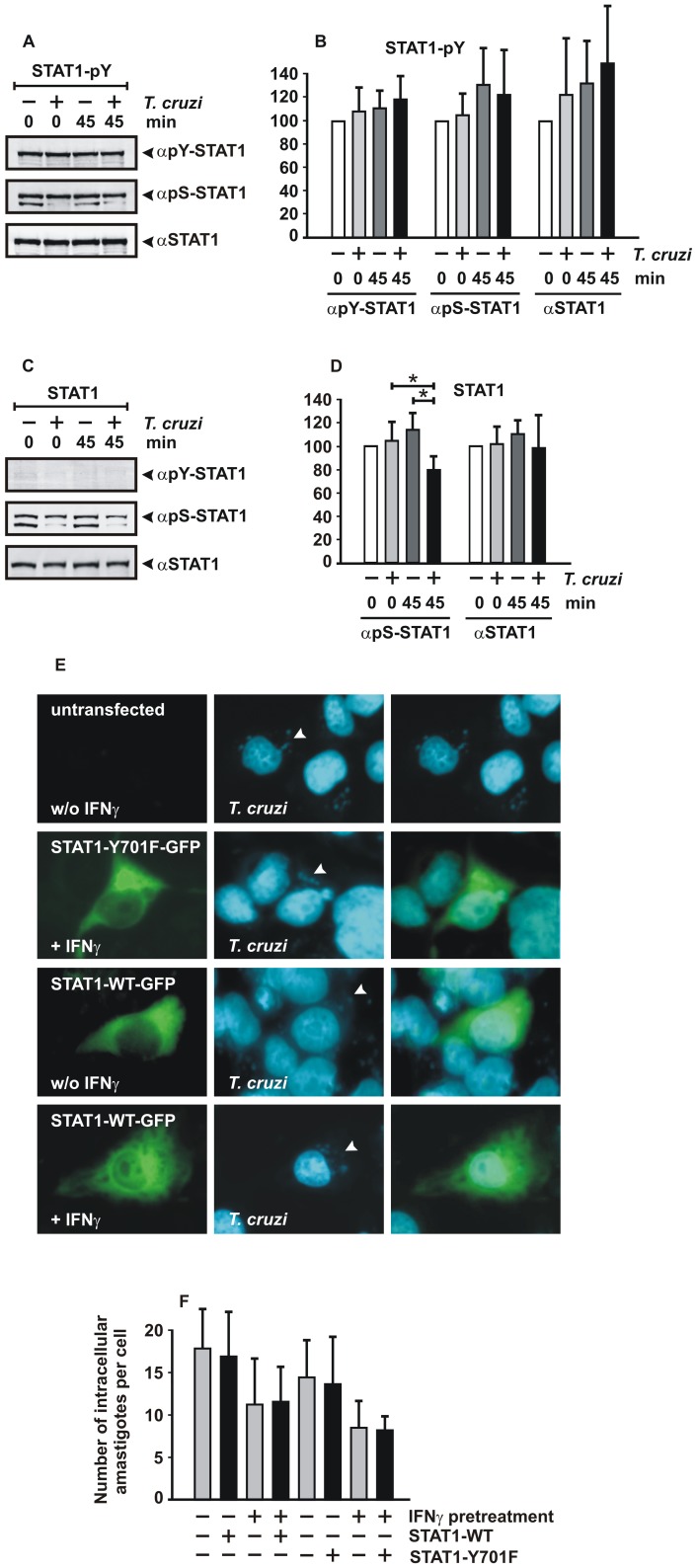
STAT1 serine phosphorylation is impaired in *T. cruzi*-infected cells. *In vitro* dephosphorylation assays were performed by incubating equal amounts of cell extracts from STAT1-GFP-expressing U3A cells (20 µl) with cell extracts from uninfected or parasite-infected STAT1-negative U3A cells. Co-incubation of the lysates lasted for 0 min and 45 min at room temperature, respectively. Before cell lysis, the U3A cells expressing the marker protein STAT1-GFP had either been treated with IFNγ (A,B) or left untreated (C,D). The reactions were then loaded on SDS polyacrylamide gels and tested for the amount of phosphorylated and total STAT1 by means of Western blotting using the antibodies indicated. Representative Western blots (A,C) and a quantification of these results (B,D) are shown. Arrowheads at the right-hand margin of the membranes mark bands corresponding to STAT1. Note that the faster-migrating band labeled with the anti-phospho-S727 antibody is an unknown non-STAT1 protein, whose expression is typically reduced in extracts from parasite-infected cells. The experiment was repeated at least four times with similar results. (E) Detection of intracellular parasites in STAT1-reconstituted U3A cells. Cells expressing a GFP fusion of wild-type STAT1 or the tyrosine-phosphorylation-deficient point mutant Y701F were treated for 6 h with 5 ng/ml IFNγ or left untreated, as indicated. Subsequently, cells were infected for 18 h with parasites at an MOI of 2, and parasitic and human nuclei were stained in fixed cells with Hoechst dye. Adjacent non-transfected cells were used as control. Arrowheads mark localization of parasites in the cytoplasm of human cells. The experiment was performed twice for each condition with similar results. (F) Quantification of results from (E) in at least n = 14 cells per sample showing that STAT1-GFP expression did not prevent replication of amastigotes.

Finally, we wondered whether the presence of intracellular *T. cruzi* counteracts the parasite-killing action of phosphorylated STAT1 by directly targeting the STAT1 molecule. Given our observation that STAT1-mediated IFNγ signaling represents a critical component in the protection against the parasite, we first assessed whether *T. cruzi*-infected cells circumvent IFNγ-stimulated anti-parasitic activity through abnormal dephosphorylation of STAT1. For this purpose, we incubated equal amounts of extracts from *T. cruzi*-infected or uninfected U3A cells with extracts from uninfected U3A cells expressing a recombinant fusion of green fluorescent protein with STAT1 (STAT1-GFP). The GFP fusion instead of untagged STAT1 was chosen as substrate, since from our previous experiments, as described above, it was known that parasite infection resulted in an increased expression of endogenous STAT1. The GFP-tagged STAT1 was readily distinguishable from its untagged native counterpart due to the presence of a 27 kD GFP domain. Tyrosine-phosphorylated and non-tyrosine-phosphorylated STAT1-GFP was obtained separately from IFNγ-pre-treated and untreated U3A cells, respectively. The STAT1-GFP marker protein was then incubated *in vitro* for either 0 min or 45 min with cellular extracts from non-infected and infected cells. Immunoblotting results using tyrosine-phosphorylated STAT1-GFP showed that neither tyrosine 701 nor serine 727 phosphorylation was modulated when reacted with cellular extracts from *T. cruzi*-infected cells (Fig. 5A,B). Thus, we found no evidence that amastigotes replicating in the cytoplasm inhibit the establishment of an IFNγ-inducible anti-parasite state through secreting effector molecules that directly affect tyrosine-phosphorylated STAT1.

However, different results were obtained when we substituted non-phosphorylated STAT1-GFP for tyrosine-phosphorylated STAT1-GFP. By incubating non-phosphorylated STAT1-GFP with extracts from *T. cruzi*- or mock-uninfected U3A cells, we unexpectedly found significantly reduced amounts of serine-phosphorylated STAT1 in *T. cruzi*-infected cells as compared to those in uninfected cells (Fig. 5C,D). This finding suggested that a component in the lysates of parasite-infected cells had reduced exclusively the level of serine-phosphorylated, but not tyrosine-phosphorylated STAT1-GFP marker protein during the 45 min of *in vitro* incubation.

Finally, we wondered whether *T. cruzi* parasites replicate in STAT1-reconstituted U3A cells. For this purpose, we transfected STAT1-negative U3A cells with expression plasmids coding for either wild-type STAT1-GFP or its tyrosine-phosphorylation-deficient point mutant Y701F. Twenty hours after transfection, STAT1-GFP-expressing cells were treated for 6 h with IFNγ (5 ng/ml) or left untreated. Cells were then infected with parasites at an MOI of 2, and 18 h later in fixed cells intracellular parasites were stained with Hoechst dye. As shown in Fig. 5E and F, amastigotes were readily detectable in the cytoplasm of non-transfected cells as well as adjacent STAT1-GFP-expressing cells, irrespective of whether or not STAT1 was functional in IFNγ signaling.

## Discussion

Subversion of innate host immune responses, particularly those induced by the pro-inflammatory cytokine IFNγ, is increasingly recognized as a key feature that contributes to the success of obligate intracellular protozoan microorganisms which after invasion of warm-blooded animals cause a persistent life-long infection [43]. Anti-microbial effector mechanisms by which IFNγ confers resistance to intracellular parasitic pathogens are best studied in the model organism *Toxoplasma gondii* [29], [43]. Among other mechanisms, including killing by iNOS and IDO, the IFNγ-induced p47 GTPases have been placed at the centre of the initial defense against invading *Toxoplasma gondii* parasites, since shortly after infection they facilitate the disruption of the parasitophorous vacuole [44]. However, much less is known about the components of the IFNγ signal pathway that combat the etiologic agent of Chagas disease, and, conversely, how *T. cruzi* counteracts this attack [6], [12], [13], [16], [19], [20].

In the present study, we revealed the crucial role of STAT1 in reducing the susceptibility to *T. cruzi* infection and, in addition, identified the infection-regulated *ido* gene responsible for parasite killing to be under the transcriptional control of STAT1. Our data demonstrate that IFNγ pre-treatment causes *T. cruzi*-infected cells to enter an anti-parasitic state through the activation of the transcription factor STAT1. While the replication rate of parasites in STAT1-expressing cells was substantially reduced upon IFNγ stimulation, no such effect was observed in U3A cells deficient in STAT1 expression (Fig. 1). There are reports in the literature showing that cytokines synthesized during *T. cruzi* infection, including IFNγ, stimulate infected cells in an autocrine manner [6], [45]. In HFF cells, the duration of pre-treatment with IFNγ is pivotal for resistance against the parasite, as measured by the numbers of infected cells and intracellular parasites. An 18-hour exposure to IFNγ significantly increased the amount of non-phosphorylated STAT1 molecules in uninfected HFF and A549 cells (Fig. 2A), which was not surprising given the fact that tyrosine-phosphorylated STAT1 up-regulates its own gene in a positive feedback loop (Fig. 3C). Stark and colleagues reported that the newly synthesized, non-phosphorylated STAT1, which persists for days after IFNγ priming, functions as a key transcriptional regulator for a subset of genes by mechanisms distinct from those used by tyrosine-phosphorylated STAT1 (for a review, see [42]).

The induction of non-phosphorylated STAT1 as a secondary transcription factor may be an element of a more general strategy to combat intracellular parasites by circumventing the potentially harmful action of cytokine-driven STAT1 activation. Best studied, albeit not in parasite infection, is the regulation of the IFNγ-inducible *lmp*2 gene, which encodes a component of the 20S proteasome (*l*ow *m*olecular mass *p*olypeptide *2*) and requires a complex consisting of non-phosphorylated STAT1 and interferon-regulatory factor 1 for promoter binding and basal transcription [46]. Thus, it will be interesting to know whether, also in *T. cruzi*-infected cells, non-phosphorylated STAT1 acts as a transcriptional regulator by interacting with other cofactors or *cis*-acting elements.

In our study we confirm that, even in the absence of any exogenous IFNγ added to the cells, infection with the highly pathogenic Y strain of *T. cruzi* results in a robust increase in the amount of cellular STAT1 [26]. The induction of STAT1 was observed in HFF and A549 cells both at the mRNA and protein level (Fig. 3A and 2A-C) and was associated with increased binding of STAT1 homodimers to GAS elements (Fig. 2D-G). Moreover, we found that infection of HFF cells and A549 cells with the parasite leads to phosphorylation of the critical tyrosine 701 and serine 727 residue (Fig. 2B), both of which are required for maximal transcriptional response to IFNγ [34]–[37]. These findings demonstrate a profound cellular response to infection with *T. cruzi*, albeit intracellular parasites could not be cleared and parasite multiplication successfully continued even in STAT1-expressing cells (Fig. 5E,F).

Our findings in HFF fibroblasts and A549 epithelial cells differ from a previous study in murine macrophages infected with *T. cruzi*, in which Bergeron and Olivier failed to detect tyrosine phosphorylation or GAS binding activity of STAT1 [27]. While these authors reported that in murine macrophages there is no evidence that the JAK2/STAT1 signaling pathway accounts for the observed *T. cruzi*-mediated NO production, we demonstrate here a significant up-regulation of *inos* mRNA in response to intracellular replicating *T. cruzi* parasites in two different cell lines, as determined by real-time PCR (Fig. 3A). However, infection with *T. cruzi* outranged the elevated *inos* mRNA synthesis as compared to IFNγ stimulation of cells. Additionally, we found that the amount of *inos* mRNA rises with increased MOI (Fig. 3C). Similar observations were made for the *ido* and *stat1* transcripts but not for a control gene, suggesting that the transcriptional regulation of these three and the other known STAT1-regulated target genes tested (*mig1*, *gbp1*, and *irf1*) is dependent on tyrosine phosphorylation of STAT1.

**Figure 6:**
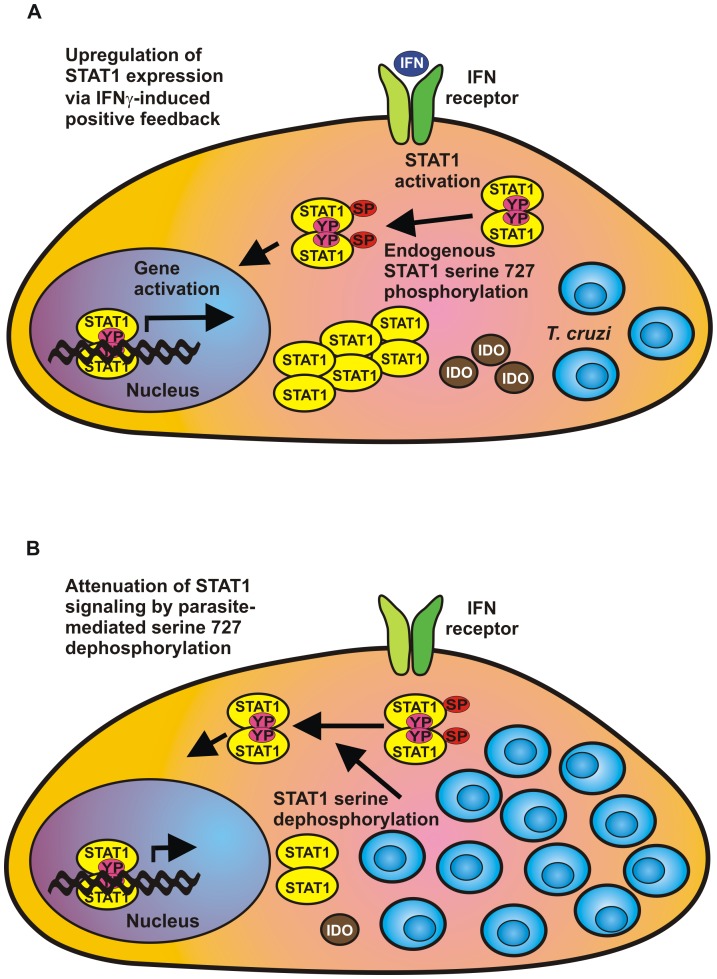
STAT1-regulated suppression of *T. cruzi* replication and its inhibition by amastigotes. The model depicts two phases in host-parasite interactions: (A) Induction of STAT1 expression by phosphorylated STAT1 via a positive feedback loop which resulted from the stimulation of cells with IFNγ. (B) Inhibition of STAT1 transcriptional activity by *T. cruzi*-mediated dephosphorylation of serine 727. Serine dephosphorylation of STAT1 leads to blunted indoleamine 2,3-dioxygenase (IDO) expression as part of a parasitic defense mechanism. The established autocrine production of interferons through a TLR-dependent pathway in the parasite-infected cells is not depicted in the figure.

Despite the activation of STAT1 and its downstream genes, we found signs of ongoing intracellular multiplication of the parasite and subsequent cell lysis. This observation suggests an evasion mechanism of *T. cruzi* from the cellular innate immune system. In our *in vitro* dephosphorylation assays using GFP-tagged STAT1 as an artificial substrate, we demonstrated that intracellular *T. cruzi* amastigotes counteract the microbicidal effects of STAT1 by directly targeting serine-phosphorylated STAT1. The presence of a dephosphorylating activity in extracts from *T. cruzi*-infected cells was restricted to STAT1 molecules phosphorylated on serine 727, while there was no depletion of tyrosine-phosphorylated STAT1 triggered by the interaction between the pathogen and the host cell. These data support the hypothesis that *T. cruzi* parasites have evolved effective ways to repress STAT1 signaling by directing newly synthesized serine-phosphorylated STAT1 molecules to either dephosphorylation or proteosomal degradation. The fact that we observed no apoptotic cell death following *T. cruzi* infection suggests that the diminution of STAT1 serine phosphorylation is not a result of general cell stress or specific phagocytosis, but rather accounts for an effective strategy whereby amastigotes can subvert microbicidal activity, most probably by directly impairing the serine phosphorylation status (Fig. 6). Since it has been reported that serine residue 727 is essential for the induction of apoptosis [47], [48], we hypothesize that *T. cruzi*-mediated serine dephosphorylation may prevent apoptotic cell death of infected cells and contributes to the long-term survival of the parasite.

Our observation is reminiscent of the action of *Leishmania donovani* in the proteasome-mediated degradation of STAT1 in macrophages [49], [50] or of paramyxovirus-encoded proteins that degrade STAT1 by recruiting a cellular E3-ubiquitin-protein ligase for specific proteolysis [51]–[53]. Given that STAT1 serine dephosphorylation occurred in cell-free extracts within 45 min of incubation, it is unlikely that this inhibitory impact on the IFNγ/STAT1 signal pathway in *T. cruzi*-infected cells is a consequence of transcriptional responses such as an up-regulation of SOCS (*s*uppressor *o*f *c*ytokine *s*ignaling) proteins.

In summary, our data demonstrate that the STAT1 signal pathway contributes to the IFNγ-induced anti-parasitic state in *T. cruzi*-infected human cells. The protective effect of STAT1 activation results in part from the induction of the *ido* gene, whose gene product catalyzes tryptophan depletion and inhibits growth of the intracellular amastigotes. However, once intracellular, the parasite keeps on replicating, despite the expression of activated STAT1 and its downstream genes. Obviously, the cellular response is insufficient to impair further parasite propagation, suggesting a parasitic evasion strategy. In line with this assumption, we show that *T. cruzi* amastigotes replicating intracellularly antagonize STAT1 signal transduction by promoting selective dephosphorylation of serine 727. Serine phosphorylation has been well established as a prerequisite for maximal transcriptional activation, and our observations underscore the notion that post-transcriptional modification of STAT1 is the target for the counter-attack by the parasites to override this protective function. These findings suggest that the control of the IFNγ/STAT1 pathway has evolved as an important issue in the fight between *Trypanosoma cruzi* and the host for supremacy.

## Supporting Information

File S1 Contains the following files: **Figure S1**. The inhibitory effect of IFNγ on parasite replication requires cytokine pre-treatment. (A,B) Twelve hours of pre-treatment with interferon-γ significantly decreased parasite load, as determined by reduced numbers of both infected cells (A) and intracellular amastigotes per cell (B). HFF cells were either left untreated (-IFNγ, black columns) or treated with 5 ng/ml IFNγ (+IFNγ, grey columns) before being infected with *T. cruzi* trypomastigotes at an MOI of 2 and 10, respectively (n = 2 in duplicate). (C,D) The inhibitory effect of IFNγ on parasite replication requires cytokine pre-treatment of cells and is not observed when cells are exposed simultaneously to IFNγ and parasites. Equal cell numbers of HFF cells were left untreated without IFNγ (black column), pre-treated for 6 h with IFNγ before parasite infection (dark grey columns) or simultaneously exposed to IFNγ and the indicated parasites strain (light grey columns). All cells were infected with *T. cruzi* at an MOI of 5, and 20 h post-infection numbers of infected cells (C) and intracellular amastigotes (D) per microscopic field were counted in each sample (n = 2 in duplicate). **Figure S2**. Long-time exposure of HFF cells to IFNγ resulted in up-regulation of endogenous STAT1 expression. Cells were stimulated for 0 h, 6 h or 12 h with 5 ng/ml IFNγ in the presence or absence of *T. cruzi* infection (MOI 20), as indicated. In lane 5, lysates from cells pre-treated for 4 h with IFNγ following by 8 h of simultaneous exposure to IFNγ and *T. cruzi* were loaded on the gel. A representative immunoblot result using phospho-tyrosine-specific STAT1 and pan-STAT1 antibodies is shown (n = 2). **Figure S3**. *Trypanosoma cruzi* infectious-dose-dependent increased levels of *gpb1*, *ido*, *irf1*, and *stat1* gene expression in A549 cells, as determined by real-time PCR assays. Histograms depict levels of gene activation after infection of cells with parasites at MOIs of 0, 5 and 10, respectively. *Adam19* was used as a negative control. Data are normalized to the expression level of the housekeeping gene *gapdh* and presented as means and standard deviations.
(DOC)
