# Supplementary material for: Trypanosoma cruzi Evades the Protective Role of Interferon-Gamma-Signaling in Parasite-Infected Cells
Source: PLoS One. 2014 Oct 23;9(10):e110512. doi: 10.1371/journal.pone.0110512 (PMC4207753; doi:10.1371/journal.pone.0110512)

**Supplemental data**

List of primer pairs used for RT-PCR:

GBP1F, 5´-GGTCCAGTTGCTGAAAGAGC-´3;

GBP1R, 5´-TGACAGGAAGGCTCTGGTCT-´3;

IRF1F, 5´-AGCTCAGCTGTGCGAGTGTA-´3;

IRF1R, 5´-TAGCTGCTGTGGTCATCAGG-´3;

MIG1F, 5´-CCACCGAGATCCTTATCGAA-´3;

MIG1R, 5´-CTAACCGACTTGGCTGCTTC-´3;

IDOF, 5´-GATGAAGAAGTGGGCTTTGC-´3;

IDOR, 5´-CGCTGTGACTTGTGGTCTGT-´3;

INOSF, 5´-ACAAGCCTACCCCTCCAGAT-´3;

INOSR, 5´-TCCCGTCAGTTGGTAGGTTC-´3;

NFKBF, 5´-GGAAACCATATGAGCCAGAG -´3;

NFKBR, 5´-CTCATAGTTGTCCATAAGTG-´3;

ADAM19F, 5´-CCCTTTCCCAAAGTGTTCAA-´3;

ADAM19R, 5´-GCAGCAGGGGTTGTTACATT-´3;

GAPDHF, 5´-GAAGGTGAAGGTCGGAGTC-´3;

GAPDHR, 5´-GAAGATGGTGATGGGATTTC-´3;

STAT1F, 5´-CCGTTTTCATGACCTCCTGT-´3; and

STAT1R, 5´-TGAATATTCCCCGACTGAGC-´3.

**Supplemental figures**

**Supplemental Figure 1:** (A,B) Twelve hours of pre-treatment with interferon-γ significantly decreased parasite load, as determined by both reduced numbers of infected cells (A) and numbers of amastigotes per cell (B). HFF cells were either left untreated (-IFNγ, black columns) or treated with 5 ng/ml IFNγ (+IFNγ, grey columns) before being infected with *T. cruzi* trypomastigotes at an MOI of 2 and 10, respectively (n=2 in duplicate). (C,D) The inhibitory effect of IFNγ on parasite replication requires cytokine pre-treatment of cells and is not observed when cells are exposed simultaneously to IFNγ and parasites. Equal cell numbers of HFF cells were left untreated without IFNγ (black column), pre-treated for 6 h with IFNγ before parasite infection (dark grey columns) or simultaneously exposed to IFNγ and parasites (light grey columns). All cells were infected with *T. cruzi* at an MOI of 5, and after 20 numbers of infected cells per microscopic field (C) and numbers of intracellular amastigotes (D) were counted in each sample (n=2 in duplicate).

**Supplemental Figure 2:** Long-time exposure of HFF cells to IFNγ resulted in up-regulation of endogenous STAT1 expression. Cells were stimulated for 0 h, 6 h or 12 h with 5 ng/ml IFNγ in the presence or absence of *T. cruzi* infection (MOI 20), as indicated. In lane 5, lysates from cells pre-treated for 4 h with IFNγ following by 8 h of simultaneous exposure to IFNy and *T. cruzi* were loaded on the gel. A representative immunoblot result using phospho-tyrosine-specific STAT1 and pan-STAT1 antibodies is shown (n=2).

**Supplemental Figure 3:** *Trypanosoma cruzi* infectious-dose-dependent increased levels of *gpb1*, *ido*, *irf1*, and *stat1* gene expression in A549 cells, as determined by real-time PCR assays. Histograms depict levels of gene activation after infection of cells with parasites at MOIs of 0, 5 and 10, respectively. *Adam19* was used as a negative control. Data are normalized to the expression level of the housekeeping gene *gapdh* and presented as means and standard deviations.


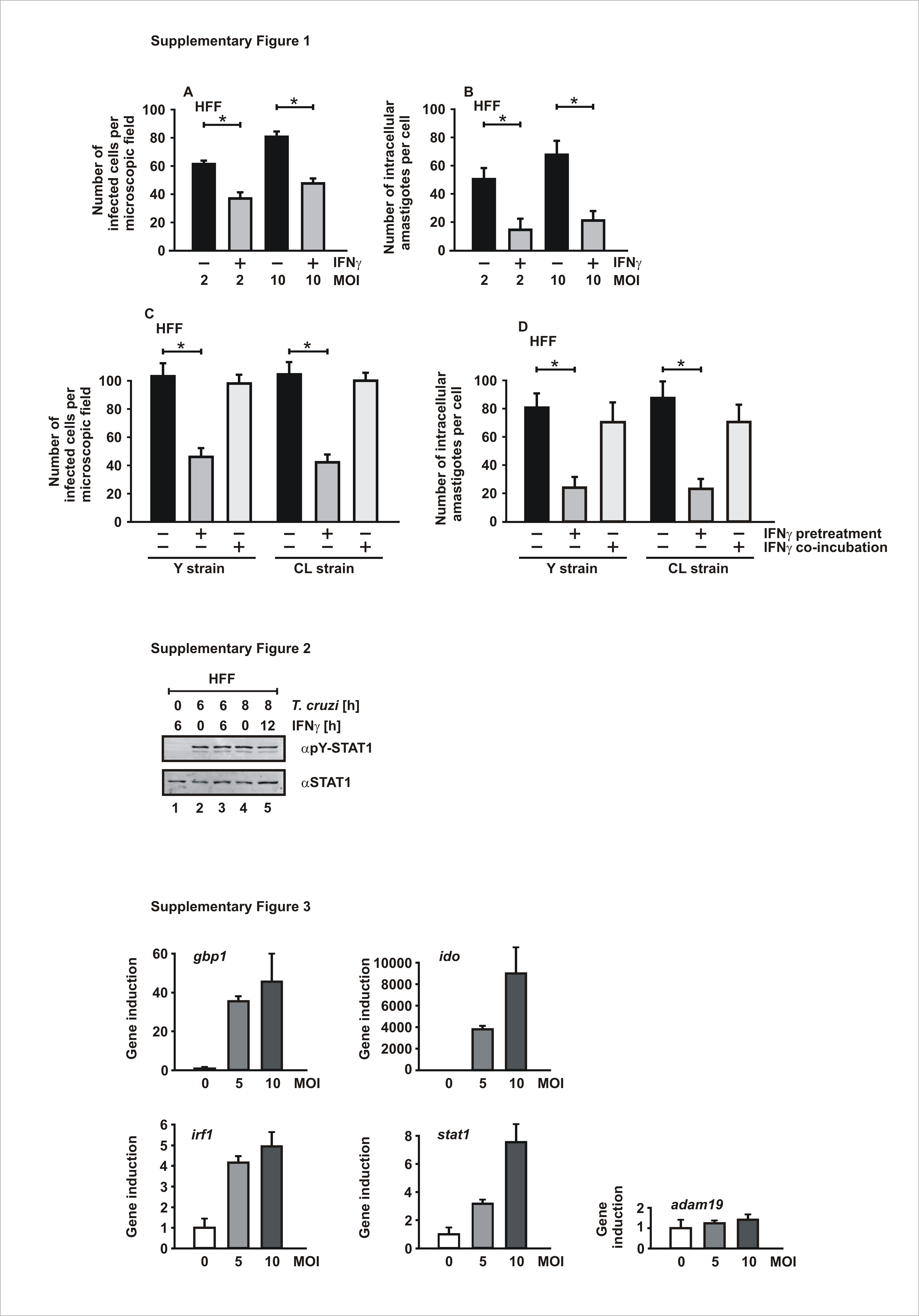

Supplement: File S1 — Contains the following files: Figure S1. The inhibitory effect of IFNγ on parasite replication requires cytokine pre-treatment. (A,B) Twelve hours of pre-treatment with interferon-γ significantly decreased parasite load, as determined by reduced numbers of both infected cells (A) and intracellular amastigotes per cell (B). HFF cells were either left untreated (-IFNγ, black columns) or treated with 5 ng/ml IFNγ (+IFNγ, grey columns) before being infected with T. cruzi trypomastigotes at an MOI of 2 and 10, respectively (n = 2 in duplicate). (C,D) The inhibitory effect of IFNγ on parasite replication requires cytokine pre-treatment of cells and is not observed when cells are exposed simultaneously to IFNγ and parasites. Equal cell numbers of HFF cells were left untreated without IFNγ (black column), pre-treated for 6 h with IFNγ before parasite infection (dark grey columns) or simultaneously exposed to IFNγ and the indicated parasites strain (light grey columns). All cells were infected with T. cruzi at an MOI of 5, and 20 h post-infection numbers of infected cells (C) and intracellular amastigotes (D) per microscopic field were counted in each sample (n = 2 in duplicate). Figure S2. Long-time exposure of HFF cells to IFNγ resulted in up-regulation of endogenous STAT1 expression. Cells were stimulated for 0 h, 6 h or 12 h with 5 ng/ml IFNγ in the presence or absence of T. cruzi infection (MOI 20), as indicated. In lane 5, lysates from cells pre-treated for 4 h with IFNγ following by 8 h of simultaneous exposure to IFNγ and T. cruzi were loaded on the gel. A representative immunoblot result using phospho-tyrosine-specific STAT1 and pan-STAT1 antibodies is shown (n = 2). Figure S3. Trypanosoma cruzi infectious-dose-dependent increased levels of gpb1, ido, irf1, and stat1 gene expression in A549 cells, as determined by real-time PCR assays. Histograms depict levels of gene activation after infection of cells with parasites at MOIs of 0, 5 and 10, respectively. Adam19 was [file pone.0110512.s001.doc]
